# Extracellular Vesicles, Cell-Penetrating Peptides and miRNAs as Future Novel Therapeutic Interventions for Parkinson’s and Alzheimer’s Disease

**DOI:** 10.3390/biomedicines11030728

**Published:** 2023-02-28

**Authors:** Cameron Noah Keighron, Sahar Avazzadeh, Katarzyna Goljanek-Whysall, Brian McDonagh, Linda Howard, Thomas Ritter, Leo R. Quinlan

**Affiliations:** 1 Physiology School of Medicine, University of Galway, H91 TK33 Galway, Ireland; 2 Cellular Physiology Research Laboratory (CPRL), School of Medicine, University of Galway, H91 TK33 Galway, Ireland; 3 Institute of Life Course and Medical Sciences, University of Liverpool, Liverpool L69 3BX, UK; 4 Regenerative Medicine Institute, School of Medicine, College of Medicine, Nursing and Health Sciences, University of Galway, H91 TK33 Galway, Ireland; 5 CURAM SFI Research Centre for Medical Devices, University of Galway, H91 TK33 Galway, Ireland

**Keywords:** Parkinson’s, Alzheimer’s, neurodegeneration, extracellular vesicles, microRNA, cell-penetrating peptides

## Abstract

Neurodegeneration is hallmarked by the progressive loss of dopaminergic neurons and/or a significant increase in protein aggregates in the brain. Neurodegenerative diseases are a leading cause of death worldwide with over 15 million people currently suffering from either Parkinson’s disease (PD) or Alzheimer’s disease (AD). PD is often characterized by both motor and non-motor symptoms, including muscle rigidity, tremors and bradykinesia, with AD displaying symptoms of confusion and dementia. The current mainstay of therapeutics includes pharmacological approaches such as levodopa to replace dopamine in PD patients, deep brain stimulation in affected regions of the brain and physical therapy. However, these treatments are typically not disease-modifying, though they do help at least for some time with symptom management. These treatments often also fail due to their inability to cross the blood–brain barrier. There is a need to develop new strategies to target neurodegeneration in an ever-ageing population. First, we review the current PD and AD treatments and their limitations. Second, we review the current use of extracellular vesicles (EVs), cell-penetrating peptides (CPPs) and miRNAs as neuroprotective agents. Finally, we discuss the possibility of exploiting these as a combinatory therapeutic, alongside some potential drawbacks.

## 1. Introduction

**Figure 1:**
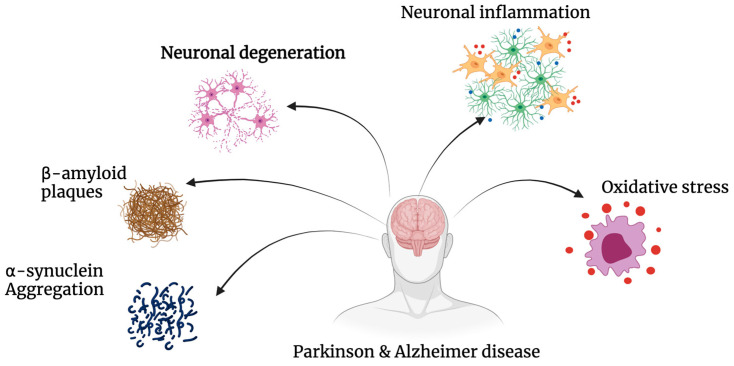
Pathogenesis of Alzheimer’s’ disease and Parkinson’s’ disease. AD and PD share common pathways of degeneration. Both neurodegenerative diseases exhibit significant amounts of oxidative stress, neuronal inflammation and degeneration, as well as the build-up of insoluble proteins including β-amyloid and α-synuclein.

The hallmark of neurodegeneration is the progressive loss of brain function, often with overlapping biological and clinical symptoms [1]. In general, neurodegenerative diseases share common pathologies and pathways, most prominently protein aggregation, oxidative stress, neuroinflammation and blood–brain barrier dysfunction, leading primarily to the death of neurons from various regions of the brain [2,3,4]. Neuronal degeneration is profound in both Alzheimer’s (AD) and Parkinson’s disease (PD) with numerous overlapping pathways implicated, such as regional aggregation of intracellular proteins Tau and Alpha-synuclein [5]; complex genotype–phenotype relationships with common mutations such as leucine-rich repeat kinase 2 (LRRK2), PTEN-induced kinase 1 (PINK1) and amyloid precursor protein (APP) [6]; and alterations in pathways such as autophagy–lysosome activity, creating an imbalance between autophagosome formation and the autophagic degradation usually involved in clearing aggregated proteins [7]. Mitochondrial homeostasis is also implicated in AD and PD, leading to neuronal cell death via an increase in oxidative stress [8,9]. Lastly, innate immunity, synaptic toxicity and network dysfunction all contribute to the neuronal loss observed in AD and PD [10] (Figure 1).

AD and PD are the two most common neurodegenerative diseases worldwide. Cognitive dysfunction is the primary symptom exhibited in AD, while significant motor dysfunction is cardinal to PD [5]. AD is the most common cause of dementia, with a prevalence estimated at 24 million worldwide, which is expected to continue to rise over the next decade (GBD 2019 Dementia Forecasting Collaborators 2022). AD affects around 11% of the population over the age of 65, with PD affecting 2–3% of the population over 65 years of age, becoming the second most common neurodegenerative disease after AD [11]. AD can be classified into four stages: pre-clinical/pre-symptomatic, mild/early stage, moderate stage and severe/late stage [12,13,14]. These stages are often characterised by progressive memory loss, impaired balance, aphasia-like symptoms and an overall lack of independence in carrying out activities of daily living [15,16,17]. Two neuropathological changes have been identified in AD; positive lesions and negative lesions. Accumulation of neurofibrillary tangles, amyloid plaques and other deposits are significant indicators of AD, while on the other hand, neuronal, neuropil and synaptic loss-induced atrophy is also indicated in AD [18,19]. While the precise cause of the underlying pathological changes in AD is unknown, risk factors including age, genetics, traumatic brain injuries, diet and immune system dysregulation are some of the key contributors [20].

PD is associated with a lack of dopamine and an overall slowing of movement (bradykinesia) along with either a resting tremor or rigidity [21]. It has been suggested that there are two stages to PD, early (1 and 2) and late (3 and 4). In the early stages, symptoms can include rapid eye movement sleep behaviour disease (including sleep paralysis), as well as decreased smell, suggesting onset in the medulla and olfactory bulb. In stages 3 and 4, symptoms are more typical of cognitive impairment, including issues with movement and gait as well as hallucinations, suggesting that this stage’s pathology has progressed to the substantia nigra pars compacta and other midbrain and basal forebrain regions [22,23], often with aggregates of alpha-synuclein [23].

## 2. Aetiology and Pathophysiology of Disease

PD and AD are characterised by the presence of insoluble protein deposits, β-amyloid plaques and tau-containing neurofibrillary lesions in AD and α-synuclein-rich Lewy bodies in PD (Figure 1). Neuropathological changes in the disease progression and pathology of AD include neurofibrillary tangles, amyloid plaques, dystrophic neurites and neuropil threads [24]. PD presents with abnormal α-synuclein aggregates and the presence of Lewy bodies with selective loss of dopaminergic neurons [25,26]. Typically, PD is diagnosed at the mid to late stage of the disease due to a long period of dormancy between the initial loss of dopaminergic neurons and the development of more “typical” clinical symptoms [21,27]. AD follows a similar pathway with initial symptoms presenting over a 2–4-year period, then progressively worsening over the subsequent 10 years. PD and AD are largely considered to be sporadic, with evidence in recent years continuing to support the hypothesis that AD and PD may have substantial genetic components. In PD, this includes reported defects in the SNCA, PINK1 genes (associated with abnormal mitochondria and increased apoptosis), parkin (associated with impaired damaged protein tagging with ubiquitin), LRRK2 (associated with increased neuroinflammation) and DJ-1 (associated with increased reactive oxygen species), with genes such as APP (associated with the generation of beta-amyloid peptides), PSEN1 and PSEN2 (both interact with APP and are associated with the overproduction of toxic beta-amyloid peptides) displaying defects in AD [28,29,30,31].

Taking a closer look at both AD and PD suggests many commonalities in their pathophysiology, with many genes shared and co-expressed [32], as well as stark differences [33]. AD is well known for its increased concentration of Aβ42 (β-amyloid 42—due to mutations in the APP gene) which encourages the production of oligomers (that are neurotoxic). These oligomers cluster and eventually form plaques that contribute to symptoms presented above for AD [34,35]. Tau, usually functioning to stabilise axonal microtubules, is abnormally phosphorylated in AD. When this occurs, these highly phosphorylated tau proteins tend to clump together into filaments and further into insoluble neurofibrillary aggregates, spreading throughout the brain [36,37]. Tauopathy is also closely linked to granulo-vacuolar degeneration (GVD) in AD patients. Granulo-vacuolar bodies (GVBs) are often present in hippocampal pyramidal cells of those with AD, which is likely associated with cognitive decline in patients [34,38,39].

The increased loss of neurons in the substania nigra pars compacta (mostly), combined with the presence of abnormal aggregates, underpins the pathophysiology of PD [40]. Losing these neurons, predominately dopaminergic neurons, reduces dopamine levels, thus leading to the symptoms [41]. Drilling down into mechanisms, alterations in α-synuclein lead to either an increase in aggregation of protein or diminished capacity for its degradation, creating fibrilization of α-synuclein in Lewy bodies or neurites promoting neurodegeneration [42,43]. Furthermore, this α-synuclein accumulation directly increases levels of mitochondrial stress and reactive oxygen species [44,45]. Parkin is associated with many subcellular compartments such as synaptic vesicles and the endoplasmic reticulum, as well as playing a neuroprotective role via mitochondria by delaying mitochondrial swelling and the activation of caspase 3 [46]. Its function is closely linked to the ubiquitin-proteasome proteolytic pathway. Parkin is an E3 ubiquitin-protein ligase that, in partnership with E2-conjugating enzymes, selects protein substrates for ubiquitylation and their subsequent degradation [46,47,48]. Oxidative stress transforms DJ-1 (which has a key role in antioxidant activities as well as in directly inhibiting α-synuclein aggregation) into a more acidic isoform which translocates to the outer mitochondrial membrane with PINK1 (located on the inner membrane of mitochondria), regulating the function of mitochondria through phosphorylation of substrates. In neurodegeneration associated with PD, one or more of these pathways is impaired; losing parkin results in the accumulation of mitochondrial substrates, exposing mitochondria to increased stress, and proteasomal impairment amplifies this effect. A reduction in ATP exacerbates the proteasomal impairment, which leads to an increase in α-synuclein accumulation [43,49,50,51,52,53] and a further increase in neurodegeneration.

## 3. Current Therapeutic Approaches

Treatment of both AD and PD has evolved over time; however, it is still true that no effective treatment for neurodegeneration exists despite extensive time, money and effort being applied to treatment discovery. Researchers have developed a number of models for investigating both diseases, and it is important to understand current treatments, how they work and ways that they can be improved.

### 3.1. Current Therapeutic Approaches—Alzheimer’s Disease

#### 3.1.1. Pharmacology-Based Therapies

Treatment options targeting AD-associated cognitive impairment have expanded over the last decades with several FDA-approved pharmacological agents being developed. The current FDA-approved treatments for AD are limited to cholinesterase inhibitors (AChEIs) and an N-methyl-D-aspartate (NMDA) receptor antagonist [54].

Targeting acetylcholinesterase (AChE) was established to be the most consistent and viable therapeutic avenue in AD with cholinergic deficits [55]. AChEIs act by blocking cholinesterase enzymes (AChE and butyrylcholinesterase (BChE)) prolonging the half-life of ACh, leading to increased ACh levels available at the synaptic cleft [56]. There are currently three AChE-focused treatments available including galantamine, rivastigmine and donepezil, associated with different time points along the life cycle of AD [55,57]. However, despite initial success with these drugs, generating new, more effective AChEIs failed in clinical trials and has been abandoned due to low efficacy or significant side effects [55]. In more recent years, research has turned to both natural AChEIs (as opposed to synthetic) and hybrids (combining multiple AChEIs together), as a mechanism for overcoming some of the previous shortcomings [58].

Increased N-methyl-D-aspartate receptors (NMDAR) in AD patients results in altered calcium signalling in the brain, causing cell death and dysfunction. Receptor antagonists designed specifically to interact with NMDARs act by reducing calcium alterations and restoration of normal physiological function, ultimately slowing the progression of AD [59,60]. As with AChEIs, these agents are also an approved treatment for PD [59].

#### 3.1.2. Surgery-Based Therapies

There have been several surgical approaches trialled for the treatment of AD, with minimal success and mostly focused on symptom management [61]. Cerebrospinal spinal fluid shunting was thought to increase cerebral blood flow, thus increasing the nutrient supply to affected areas. By doing this, nutrients could support damaged but partially functioning neurons. However, results reported were often subjective with brief follow-up intervals [62]. More recently, this work has focused on the clearance of tau and beta amyloid; however, one study showed no effect on clearance of either protein [63]. Intraventricular infusions have also been trialled as a surgical intervention, but with limited publication of results. Most often the drugs trialled were cholinergic agents such as nerve growth factors or neuroprotective factors such as monosialitetrhexosylgangioside [61]. Lastly, tissue grafts have also been investigated as potential therapeutics in AD; however, there have been few human trials published in this area and more rigorous studies would be needed [64,65].

### 3.2. Current Therapeutic Approaches—Parkinson’s Disease

#### 3.2.1. Pharmacology-Based Therapies

There have been numerous therapeutic pathways highlighted as targets in PD, which are largely designed to tackle the specific symptoms [66,67]. AChE inhibitors are also used in the treatment of PD, focusing on PD-related dementia, with extremely variable outcomes [68]. One study noted that after the addition of AChEIs there was an improvement in gait parameters, such as swing duration and gait cycle duration in people with Parkinson’s disease (PwP) [69]. A systematic review of the use of AChEIs in PD was conducted in 2014 that theorised that AChEIs could be a positive treatment option for cognitive impairment, but it would need to be balanced with some of the adverse drug reactions observed [70].

Current pharmacological approaches include levodopa, L-DOPA, L-3,4 dihydroxyphenylalanine, dopamine agonists, amantadine, apomorphine or MAO-B inhibitors like selegiline, all of which aim to short-circuit the lack of dopamine in the substantia nigra (SN) [71]. Pharmacological interventions have a complicated relationship with the disease, often producing initial positive results, but most agents only address the dopaminergic aspects of PD and leave its progressive course unaltered [71,72]. Studies have shown that more regular and targeted dosing is required after 3–5 years of the disease and the progressive loss of independence, dyskinesia, confusion and hallucinations [73,74]. There are also reported side effects associated with pharmacological interventions including oxidative stress in patients, gastrointestinal problems, nausea and peripheral oedema [71,72,75,76,77].

Memantine is a partial NMDAR antagonist and has been suggested as a potential new treatment for both AD and PD. Blockage of the NMDA glutamate receptors can normalize the glutamatergic system balance, resulting in improvements in memory and cognitive function, as demonstrated in various animal models and some small patient studies [59,78]. However, its effects appear to have more benefits overall for PD pathologies, with only modest side effects reported such as nausea and drowsiness [59].

#### 3.2.2. Surgery-Based Therapies

Deep brain stimulation offers another avenue of treatment for the improvement of both motor and non-motor symptoms in severe cases of AD and PD with a focus on reducing Lewy bodies, neurofibrillary tangles and β-amyloid 42 oligomers [79,80], yet it is still unclear which targeted brain regions yield the most beneficial effects or which will impact quality of life most [81]. In the case of AD, deep brain stimulation does not appear to be of benefit to early onset AD but did show some promising results in later stages of AD [82].

#### 3.2.3. Physical Therapies

Physical therapy approaches support patients in adjusting their life after diagnosis, including speech therapy, lifestyle changes and cognitive behavioural therapy for both AD and PD [83,84]. Physical therapy helps to improve mood, decrease aggression and improve mobility and strength [85,86].

On the horizon is a more novel therapeutic intervention, with stem cells presenting an exciting opportunity to not only repair damage associated with the loss of dopaminergic neurons but to also adjust the dopamine pathways, reduce tauopathy and tackle neurofibrillary tangles [87,88,89].

## 4. Potential Future Novel Therapeutic Interventions

While there have been additions and advances to the therapeutic arsenal for AD and PD, there are still many deficits in creating a cohesive and comprehensive treatment plan. Most treatments focus on a specific aspect of the pathology and fall victim to the inability to cross the blood–brain barrier (BBB); however, as we uncover more information about the impact of the BBB in AD and PD, researchers will be assisted in increasing the potency of drugs by utilising targeted delivery systems [90]. There are still many questions unanswered despite all the current knowledge available on the short- to medium-term efficacy of current therapeutic approaches. There is a lack of treatments that diminish the common side effects of nausea, swelling and vomiting, as well as for a targeted treatment that can focus on a specific pillar pathway in the disease architecture. Lastly, there is a need for treatments that not only restore the losses observed in AD and PD but can also prevent further damage.

Recent research has uncovered a potential positive therapeutic effect of nanoparticles for disease therapies, with many small particles including metals, C NPs and exosomes being used in the treatment of neurodegeneration [91]. Extracellular vesicles (EVs), cell-penetrating peptides (CPPs) and microRNAs (miRNAs) have been shown to have immense potential in neurodegeneration. EVs are seen as carriers for small amounts of genetic information or other small molecules delivered via local or systemic routes [92]. Over the past decade, a tremendous amount of research has highlighted the potential of EVs. They have both the potential to ameliorate disease and may be suitable as the next generation therapeutic vehicle [93]. In parallel, cell-penetrating peptides have been discovered which are short, positively charged, amino acid sequences which can penetrate cell membranes [94]. They can translocate small macromolecules such as nucleic acids across the cell membrane as CPP+ cargo complexes [95]. miRNAs have been reported to function as mediators of cell–cell communication; however, their main function is post-transcriptional gene expression regulation [96]. Furthermore, there is more research to suggest that by using CPP+ cargo complexes, miRNAs can activate genes under certain conditions [97]. There is an exciting new era in prospect by utilising these approaches, either individually or combined, and their potential novel therapeutic effect in the field of neurodegeneration. In this review, we explore the potential use and combination of extracellular vesicles, cell-penetrating peptides and microRNAs (miRNAs) as novel therapeutic interventions for treatment of PD and AD.

### 4.1. Extracellular Vesicles in Neurodegeneration

EVs are membranous nanoparticles secreted by almost all types of cells (both animals and plants) into the extracellular space [98,99,100,101]. EVs have been associated with several roles within the central nervous system, including positive effects on cell function, survival and intracellular communication and negative effects through the detrimental spread of proteins associated with neurodegeneration [100,101].

There are three major classes of EVs: exosomes, ectosomes and apoptotic bodies. Exosomes are 40–160 nm in diameter and are typically derived from both the endocytic pathway and the Golgi apparatus. Ectosomes are more diverse at 50 nm–1 µm in diameter and derived from direct liberation of plasma membrane fragments [102]. Lastly, apoptotic bodies range from 50 to 5000 nm in diameter and are generally produced from cells undergoing programmed cell death [103]. The cell membrane or Golgi apparatus (through endocytosis) fuses with endosomes that undergo inward budding [104,105], giving rise to multivesicular bodies (MVBs) [106] that contain intraluminal vesicles (ILVs). These ILVs contain cargo, for example, lipids, proteins, mRNAs and microRNAs [107], which either are degraded by lysosomes or bind to the plasma membrane for release into the extracellular space [108].

**Figure 2:**
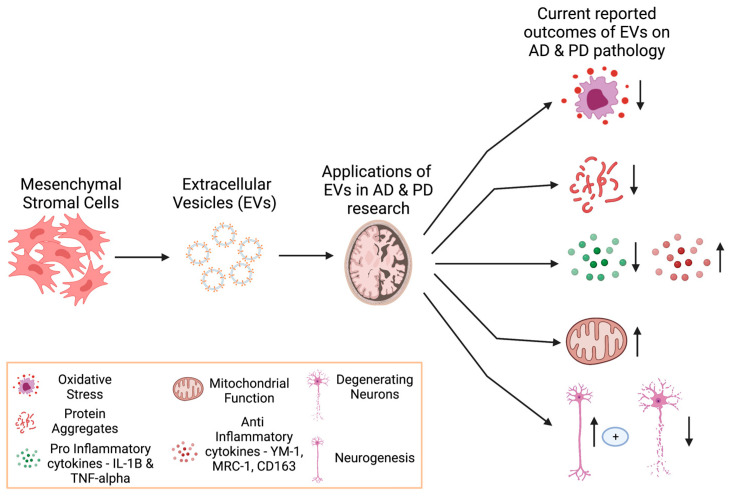
Extracellular vesicles in the therapeutics of neurodegeneration. EVs, derived from mesenchymal stromal cells (MSCs), have been studied in many AD and PD models of neurodegeneration. To date, researchers have noted a correlation between EV-based therapies and a reduction in oxidative stress, protein aggregates, pro inflammatory cytokines and degenerating neurons. In addition, they have also noted a correlation between these therapies and an increase in mitochondrial function and anti-inflammatory cytokines.

Many neurodegenerative diseases share some common features in the molecular mechanisms that underpin their pathogenesis, including protein misfolding and aggregation. The proteins that are misfolded in neurodegenerative diseases often evade usual clearing mechanisms such as refolding and degradation [109,110]. Initially, some studies suggested that EVs were carriers for misfolded or dysfunctional proteins such as α-synuclein in PD and amyloid β-protein in AD. However, this theory has been disputed over the past decade, with a broader view and the potential neuroprotective properties of these vesicles now actively being explored across many neurodegenerative diseases (Figure 2) [111,112].

Extracellular vesicles are known to have a range of positive biological functions including tissue repair, regeneration and immune surveillance [100]. Much interest in EVs has focused on their ability to modulate homeostasis, remove unwanted material and neutralise synaptic plasticity, disrupting for example the negative effects of amyloid β-protein [113]. Moreover, EVs can cross the blood–brain barrier, a shortcoming of many potential therapeutics. This is important in increasing the availability of different molecules of interest that can serve as therapeutics in neurodegeneration. Many studies have investigated the effect of EVs (derived from different sources) on features of the pathogenesis of AD and PD [114]. Mesenchymal stromal cell-derived EVs are the most common source of EVs studied to date, with researchers investigating protein aggregation, cognitive functions and neurodegenerative pathways. Protein aggregation is a major cause of pathology in both AD and PD. Over 20 studies have shown that EVs can reduce neuroinflammation, therefore leading to an associated reduction in the accumulation of Aβ and α-synuclein (SNCA) [115,116]. In addition, three studies have suggested that EVs in AD and PD models have been able to reduce oxidative stress, mitochondrial stress and pro-inflammatory cytokines such as IL-1β and TNF-a, while anti-inflammatory molecules such as YM-1, MRC1, FIZZ1, and CD163 were increased [116,117,118]. EVs have also been described as aiding neurite outgrowth and axon regeneration of damaged neurons in vivo [119,120]. Studies have also shown EVs’ ability to decrease the number/severity of damaged or destroyed neurites, both in vitro and in vivo [121,122]. There is also evidence to suggest that the overall level of apoptosis reduces after the addition of EVs in an in vitro model [123]. Lastly, it has been shown that exosomes derived from bone marrow stem cells reduced impairment associated with AD. After injection of exosomes into STZ-injected mice, researchers found that these exosomes reduced the hyperactivation of microglia and astrocytes as well as reducing IL-1β, IL-6, TNF-α, Aβ_1-42,_ and p-Tau [124].

### 4.2. Extracellular Vesicles as Cargo Delivery Vehicles

An attractive aspect of EV biology is their ability for cellular uptake as a mechanism for vesicle cargo transport. Several mechanisms of EV uptake have been proposed including direct binding to the surface of the EVs, phagocytosis, micropinocytosis and direct membrane fusion [125]. EVs can be internalized by nearly all cell types in a highly regulated process. Both the target cell and EV must possess the complimentary cell surface receptors and ligands [125,126,127].

There are a number of properties of EVs which suggest they would be effective as cargo vehicles:

1. Their ability to migrate across the BBB, in a bi-directional manner, with several studies showing their proficiency in this area, albeit with very little information about their mechanism of action [128,129,130]. A study evaluating 6-hydroxy-dopamine (6-OHDA)-induced ND on dopaminergic neurons in SH-SH5Y cells found that MSC-derived EVs reduced DA neuronal death in this in vitro model. Furthermore, they found these EVs also reduced toxicity and neuronal death in their rat model [131].
2. They can carry miRNAs. EVs can mediate the movement and delivery of many miRNAs, including miR-21. This miR is usually associated with the microglial anti-inflammatory (although in other cases it has been reported to be inflammatory) response and indicated as a potential new AD biomarker. This has been demonstrated in in vitro studies of SH-SH5Y cells that were transfected with APP [132,133].
3. EVs can be utilized as a cargo vehicle for therapeutic agents that target neurodegeneration. For example, curcumin is a drug that is suggested to reduce neuroinflammation and oxidative stress in AD, yet it lacks the ability to cross the BBB alone. It has been suggested that combining EVs and curcumin may help overcome some of these shortcomings [134,135,136]. In PD, dopamine has been trialled as a potential drug to be carried by EVs to enhance its delivery to cells, which seems to create a less transient expression of dopamine and increased neuronal function in their model [133,137]. Therapeutic proteins are loaded into exosomes ex vivo and can deliver neuroprotective effects on specific cells in the brain, with EVs crossing the BBB. They cross the BBB through targeting endothelial cells via receptor interactions, thus entering the cell through membrane fusion and endocytosis. Catalase loaded into exosomes has shown neuroprotective effects in the microglial cells, with a reduction in microgliosis in their 6OHDA-induced neurodegeneration model, as measured by a reduction in CD111b expression [138]. EVs augmented or transfected with plasmids have also been used in animal models of PD to evaluate their therapeutic effect. EVs with catalase-encoded plasmids have been shown to transverse the BBB, to incorporate with neurons and ameliorate PD symptoms in mouse models [138]. Macrophages that were genetically modified to express glial-derived neurotrophic factor (GDNF) were found to express modified EVs with GDNF and were shown to slow the progression of PD via a reduction in neuro-inflammation [139].

Despite the relative ease of transfer, the size (for example, catalase encapsulated into an EV sized between 100–200 nm showed a reduction in neuroinflammation, but other sized EVs did not show the same catalase uptake rate [138,140]) and the morphology of the EVs are crucial to success and therefore must be carefully selected [138]. It is also important to consider the origin of EVs, as this will dictate some of the neuroprotective effect they may elicit. For instance, macrophage-derived EVs innately target inflamed tissue within PD and AD, suggesting that they may be the best target EV to combine with a therapeutic protein, drug or RNA to tackle neuroinflammation in the brain [141]. There are several components to consider when exploiting EVs as therapeutics in ND.

### 4.3. Cell-Penetrating Peptides (CPPs) in Neurodegeneration

CPPs are short basic amino acid sequences that facilitate the movement of molecules, mainly proteins into cells and across the blood–brain barrier. They are typically 4–50 residues and are categorised by their physicochemical properties as cationic, amphipathic, or hydrophobic [142,143,144].

CPPs are classified based on the origin of the peptide (synthetic, chimeric or protein-derived) and their physicochemical properties (cationic, hydrophobic or amphipathic) [95]. Most CCPs are classified as cationic due to the presence of a positive charge [95]. There are three possible factors which influence CPP internalisation across membranes: the peptide concentration, the peptide sequence and the lipid components of each membrane [145,146]. Direct membrane penetration occurs at higher peptide concentrations, whereas at lower concentrations internalization occurs through endocytosis [145]. Arginine-rich CPPs yield higher concentrations in bio membranes because of their higher positive charge ratio [95]. Proteoglycans provide the first point of contact between CPPs and the cell surface via electrostatic interaction [145]. This contact initiates selective activation of some GTPases [147] influencing cell membrane fluidity and allowing CPP cell entry [145,148].

**Figure 3:**
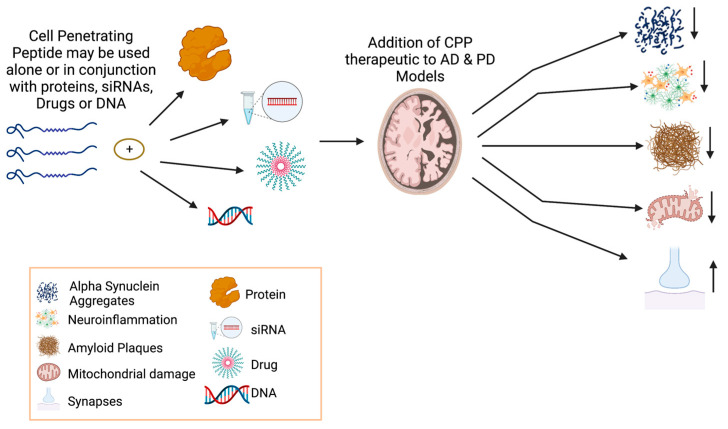
Cell-penetrating peptides as therapeutics for neurodegeneration. CPPs, either by themselves or in conjunction with proteins, siRNAs, drugs, or DNA, are thought to have many neuroprotective features in AD and PD models of neurodegeneration. When studied, CPP-based therapies reduce protein aggregates, neuronal inflammation and mitochondrial dysfunction while also restoring functional synapses.

To date, a limited number of studies have been performed on the neuroprotective and other beneficial effects of CPPs in AD and PD (Figure 3). For example, gastric inhibitory polypeptide (GIP), a hormone and growth factor playing a role in the brain, has exhibited neuroprotective effects in AD and PD animal models [149]. In addition to the neuroprotective effects of GIP and other similar peptides, including glucagon-like peptide 1 (GLP), the synaptic plasticity was retained and cell repair and mitochondrial functions were restored with the preservation of dopaminergic neuronal function [150,151]. A newer approach with CPPs is engineering CPP antagonists to combat proteins of interest, including amyloid-β [152]. Researchers are additionally exploiting the relative biocompatibility and low cost of CPPs with specific therapeutic effects and efficient delivery [153]. To date studies have had success in using CPPs to inhibit AB oligomerization and neurotoxicity, thus reducing mitochondrial stress, oxidative stress and proteasomal inhibition [142].

### 4.4. Cell-Penetrating Peptides (CPPs) as Cargo Delivery Vehicles

Intracellular delivery of large molecules suffers from a reduced capacity to cross the BBB and then retention in the brain. However, CPPs have been developed to be conjugated with small molecules such as drugs due to their high affinity for intracellular delivery [154].

CPPs have been shown to efficiently enter cells with a low level of cytotoxicity [155,156], supporting their use for intracellular delivery of cargo [157]. This can help to alleviate not only the limitations involved in conventional drug delivery [157] but also facilitate the delivery of siRNAs and other molecules to the target site [156].

CPPs can facilitate intracellular delivery of proteins through covalent bonds with their cargos. CPPs conjugated with penetrating and TAT show elevated abilities to block caspase activation, important in reducing apoptosis and protecting neurons [157,158]. Tagging or encapsulating enzymes with CPPs aids their movement across the BBB for tackling effects of neurodegeneration including oxidative stress and autophagy. TAT, along with glyoxalase, catalase and CPP with L-asparaginase can prevent oxidative stress and damage to neuronal cells [159]. However, there are concerns over cationic ions that may bind with negatively charged molecules, leading to aggregation, the very thing researchers are trying to avoid in ND, leading to a pivot towards using new approaches like microRNA [160].

### 4.5. Micro RNAs (miRNAs) in Neurodegeneration

miRNAs are small non-coding RNAs discovered in the early 1990s. They are transcribed from DNA sequences in primary miRNAs, which are then further processed into pre-cursor miRNAs and mature miRNAs [96,161,162]. To date, miRNAs have been uncovered in all animal models, with recent discovery of their roles in gene expression regulation [163,164,165,166,167]. Mostly, miRNAs suppress gene expression by interacting with the 3′ UTR region of a target mRNA, but some will interact with the 5′ UTR, coding sequence or gene promoter region of other mRNAs [163,168]. In some cases, and under certain conditions (for example, with miR369-3p, TNF alpha is recruited into specific microRNAs to promote activation of specific G0 expressed mRNAs), miRNAs can induce gene expression [96,169].

miRNAs have many important roles to play in a range of biological processes. Specific neuronal miRNAs can control neuronal differentiation, excitability and function [170]. miRNA biogenesis is a tightly controlled process regulated at multiple levels. This includes transcription, RNA editing and methylation, adenylation and RNA decay [163]. The 3′ untranslated region (UTR) of mRNAs generally contains miRNA-binding sites, while the 5′ UTR is crucial for target recognition [163]. More than 60% of human protein-coding genes have at least one miRNA binding site, indicating that they may be controlled, to a certain extent, by miRNAs [171]. While this may give us an insight into the pathogenesis of neurodegenerative diseases, it also gives us a therapeutic avenue to explore. miRNAs provide a pathway to study the commonalities in the pathophysiology of both AD and PD. Dysregulated miRNAs in both diseases are primarily associated with apoptosis and inflammation, but miRNAs targeting the regulation of APP, L1CAM and the caspase family could see a reduction in neurodegeneration, as they are some common targets for dysregulation in both AD and PD [172].

Detecting both AD and PD in early stages is problematic, but miRNAs present an alternative approach to early detection, and one which is both cost-effective and non-invasive [173]. Both cerebrospinal fluid (CSF) and blood have been suggested as fluids for biomarker testing, with various studies evaluating these in the context of early detection of AD and PD [173,174]. Some examples include miR-23a and 29b, found in serum and CSF, respectively, being used to detect mild cognition impairment AD [175,176], and changes in circulating miRNAs such as miR-30a-5p and miR-185-5p being used for PD [177,178].

Many miRNAs have been indicated as positive modulators in ameliorating pathogenesis in AD and PD; for example, overexpressing miR-34a has been indicated to reduce tau synthesis in AD by binding to the 3′ UTR of human tau mRNA [179]. There is in vitro and in vivo evidence that miR34b/c regulates DJ-1 and parkin, two key PD genes, as well as for the role that miR-29 plays in neuronal protection [180,181]. With all of this in mind, either replacement miR treatment (to augment the downregulation of key miRNAs in AD and PD), or addition of neuroprotective miRNAs, may unlock new avenues for tackling neurodegeneration. Recently, miRNA 124-3p-enhanced extracellular vesicles have been shown, in vivo, to protect neurons in the substantia nigra, and they have the potential to reduce key pathophysiological problems associated with neurodegeneration such as oxidative stress, mitochondrial stress and superoxide production [182]. Targeting α-synuclein is a major therapeutic target for PD, but also for other cases of neurodegeneration. Many miRNAs have been investigated for their role in the homeostasis of α-synuclein, with several miRNAs being identified to date, including miR-7, miR-34b and miR-214, that decrease α-synuclein levels in vitro and in vivo [183]. The future of miRNAs is bright, and the possibility of exploiting their neuroprotective effects with the cargo abilities of EVs or CPPs offers a new avenue for the treatment of PD and AD.

## 5. Next Generation of Novel Therapeutics in AD and PD

Significant progress over the last decade has been made in elucidating the potential functions of EVs and CPPs mentioned above, presenting new avenues for diagnostics and therapeutics [95,184]. The research suggests that we can look at EVs and CPPs in two ways. First, they can function as intracellular carriers for therapeutics due to their ability to cross the blood–brain barrier [157,185,186,187]. Second, they could function as the next generation of therapeutics on their own [95,149,188,189].

**Figure 4:**
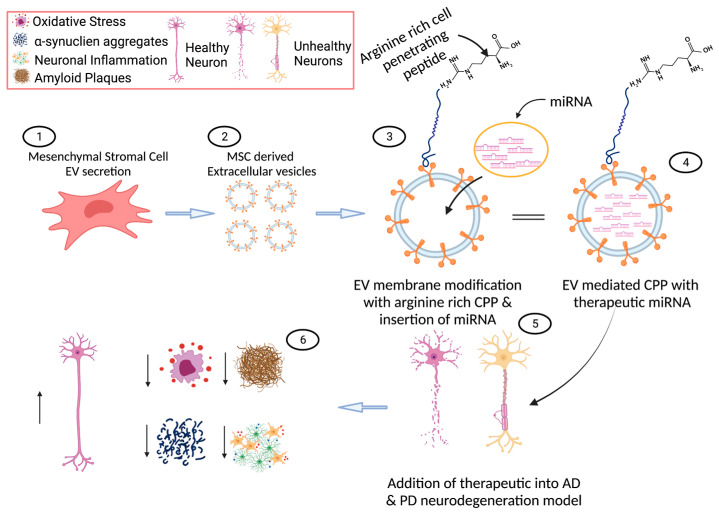
A novel approach to neurodegeneration therapeutics.

However, to look at EVs and their intracellular carrier ability, we also have to look at their ability to carry disease [190]. It has been theorised that EVs can potentiate and propagate neurodegenerative pathology via intracellular communication and transport, as well as play a role in the misfolding of key proteins associated with neurodegenerative diseases [101,191,192]. Despite this, exploiting this very mechanism in a combinatory approach with both CPPs and miRNAs could unlock novel neuroprotective therapies (Figure 4).

Conventional methods of drug delivery often incorporate liposomes or synthetic carriers; however, extracellular vesicles have many advantages over these classical methods [187]. EVs are comprised of a mixture of lipids and membrane proteins [193], facilitating the targeting of specific tissues, as well as minimising non-specific interactions [187,193,194]. There is extensive research that supports the cross-talk ability of EVs (with other cells), and the impact this may have on their therapeutic properties. Neurons and microglia communicate bidirectionally, sensing and responding to stimuli around them, and this communication can be mediated by soluble factors such as EVs [195]. The inherent ability of EVs to target specific cells allows the delivery of functional RNA and soluble cytokines involved in the immune response across the blood-brain barrier [196,197]. EVs play roles in many neuronal homeostatic processes such as neuron excitability, synaptic plasticity and microglial activation, often due to their unique intracellular communication pathways and the extensive list of potential cargo molecules [198]. We theorise that modifying the CPPs on the surface of an EV to become more arginine rich (increasing their cell-penetrating capacity), in combination with miRNAs targeting ND (such as miR-7 that targets alpha-synuclein regulation), will increase the neuroprotective effects that EVs, CPPs and miRNAs can have individually, as shown in Figure 4.

This opens an exciting domain in neurodegenerative therapeutics with the possibility of combing EVs with CPPs and miRNAs, exploiting the natural cargo delivery ability of EVs. miRNAs are small non-coding molecules that can regulate gene expression by directing mRNA cleavage or translational inhibition [199,200]. Up to one third of human genes may be regulated by miRNAs, playing roles in the immunological response to several disease pathologies [199]. Therapies involving miRNAs fall into two categories: miRNA inhibition of disease-specific miRNAs and miRNA replacement to encourage disease-free (repressed) miRNA expression [201]. In both PD and AD, dysregulation of non-coding RNAs has been reported [202,203,204]. For example, SNCA dysregulation is a major pathological problem in PD, with several miRNAs being investigated and suggested as potential α-SYN regulators, with studies demonstrating that some miRNAs could protect different cell types from neurodegeneration arising from synucleinopathy [205,206]. These include miR-7 and miR-153 protecting SH-SY5Y cells, differentiated human progenitor ReNcells, ventral midbrain (VM) cells and primary mouse neuron from degeneration from known PD-induced cell lines with MPP+ [207,208]. In AD, miRNAs have also been shown to provide a much more comprehensive approach to diagnosis. miR-222, miR-29c-3p and miR-19b-3p are much clearer biomarkers that can be detected in the biofluid of AD patients [209,210,211,212]. Furthermore, the use of miRNAs as a treatment option for AD is a continually evolving field with microarray analysis in Tg2576 transgenic mice identifying both miR-200b and miR200c as down regulators of Aβ secretion by modulation of mTOR [213].

Nevertheless, there are some limitations to using miRNAs, notably in the delivery of miRNAs to target tissues with specificity. There is scope here to combine both EVs, which possess inherent cell-targeting abilities, with therapeutic miRNAs to provide restorative therapeutic options for PD and AD [214]. In addition, while there are still gaps in our understanding of the mechanism of action of EVs, it is agreed that the delivery of EV cargo creates benefits in the treatment of disease. Taking this into account, it could be theorised to combine EVs and miRNAs together to create a complementary therapeutic, one that utilises EVs’ innate ability to target cells and tissues with specificity along with miRNAs’ neuroprotective effects [214].

Similar to the case with EVs, CPPs also facilitate the intracellular delivery of a variety of bioactive cargos; however, they lack cell specificity and often have a short/limited time of action. Hence, there is a need to optimise the CPP that is optimal for a particular target of interest. Combining CPPs with specific and more capable delivery systems, such as EVs, will improve their clinical enhancements [95,157]. There are some concerns about cytotoxicity related to both CPPs and the “cargo” they may carry—however, data indicates that cytotoxicity is low in their effective dose range, and heavily depends on CPP type and composition. This highlights the importance of selecting the appropriate CPP and concentration [154,155,215,216]. CPPs are limited by low cell and tissue selectivity [217]; however, like miRNAs, EVs may be a solution to this problem.

Recent studies have investigated modifying arginine-rich CPPs on the membranes of EVs as a method for improving effective micropinocytosis and increasing the uptake of cellular EVs expressing CPPs [218,219]. Dodecaborate-encapsulated EVs with modified hexadeca oligoarginine, a CPP, showed an amplification in the actin-dependent endocytotic pathway, micropinocytosis and cellular uptake of the specific EV [220]. This work also highlighted the importance of activating arginine peptide sequences for the efficient release of EV cellular cargo and improving the penetrating ability of these EVs [218]. This is important in the context of combining a triad approach to neurodegeneration where a potential EV could be modified with arginine-based peptides for the more efficient release of miRNAs that target alpha-synuclein in PD and tau in AD.

## 6. Current Barriers to Clinical Translation

There are still many challenges for the use of EVs and CPPs as cargo vehicles. This is a science in its infancy, and for EVs we lack an understanding of their mechanism of transport across the BBB, their biodistribution, their contents and their pharmacokinetic properties [133]. EVs have a unique surface make-up including protein receptors, transcription factors, enzymes, extracellular matrix proteins, lipids and nucleic acids (DNAs, mRNAs and miRNAs) that are within and on their surface [221]. The easiest method of loading therapeutics into EVs is to mix EVs with free drugs; however, it is difficult to control this loading efficiency and the drug may suffer from degradation within the host cells [222,223]. It is also thought the EVs contain some of their parent material from formation, thus reducing the space available for encapsulation of different drugs/proteins/RNA [140,224,225]. Many methods have been suggested for the incorporation of therapeutics with EVs, including electroporation, sonication, extrusion, and freeze/thaw cycles; however, these come with their own challenges of disrupting the integrity of the exosomes, deformation of EV membranes, exosome aggregation and low yield (with variability between EV batches) [109,226,227,228,229,230].

Incorporating therapeutics such as drugs, proteins and nucleic acids also depends on the cellular processes that produce EVs; it is a delicate balance of ensuring the right cargo molecule for the right EV, often producing variable results in terms of efficacy and potency in its effect [231]. We must also consider the source of EVs; even though nearly all cells produce some type of EV, some are not suitable to be drug carriers. In this instance, the surface proteins, size yield and intracavitary composition are key characteristics to evaluate when selecting the “ideal” EV for cargo delivery [232].

EVs contain proteins both within and on their surface, and many of these have been exploited in therapeutics, including an EV-based antioxidant and catalase delivery system for PD [138]; however, packaging active proteins or RNAs still remains challenging [232]. To date, there is no standardised method of EV extraction (although there are many guidelines from the International Society of Extracellular Vesicles (ISEV)), creating a bottleneck for the use of EVs as drug carriers, as none of the current approaches meets the ideal criteria for isolation, with many limiting factors including low specificity and low recovery in some cases [232,233,234,235].

Our current understanding of the pathophysiology of AD and PD, which is often neuron-centric, is a barrier to the generation of novel therapeutics. For example, targeting amyloid-beta and hyperphosphorylated tau as a therapeutic in AD shows very little clinical relevance despite extensive studies. Recently, work has shown that alterations in the gut microbiome may generate negative consequences for mitochondrial function in the body [236]. This discovery has proved an exciting avenue of research as a treatment target for AD and PD. Is amyloid-beta just “too much of a good thing”, with AD and PD driven by the suppression and/or dysregulation of mitochondria-linked glial processes that would normally dampen local inflammatory processes [237]?

Lastly, there is a lack of clinical translation for the use of EVs as cargo vehicles, and while some work has been done in this area, questions remain about the safety, potency and purity of any clinical products that may be developed [238].

There is less known about CPPs as therapeutics in comparison with EVs, but they are still a potentially exciting new approach to treating neurodegeneration, as they have the capacity to cross many barriers such as the BBB, skin and cornea. However, they also have many limitations, including transport efficiency, target specificity and CPP-cargo coupling [239]. CPPs are likely to be degraded after exposure to biological fluids such as blood, due to their peptidic nature, making the development of such therapeutics difficult [240]. Tracking CPPs is also tricky; it is possible to conjugate them with a fluorophore (GFP, RFP or others), but ultimately this will change the physiochemical properties of the CPP of choice and thus its biodistribution. Detecting non-labelled CPPs is hampered due to a lack of specific antibody availability for techniques such as an enzyme-linked immunosorbent assay [241], while there are also challenges around detecting fluorescently labelled CPPs in vitro, because the fluorescence may be blocked by tryptophan residues of close transmembrane proteins on the membrane bilayer [242].

CPPs are significantly limited by their chemical instability; they are subject to degradation via intra- and extracellular enzymes, subsequently lowering their concentration and thus their potency (although some biodegradability is desirable as it improves biocompatibility and patient safety) [243]. There are many strategies aiming to overcome this excessive degradation, including modifying the amino acid residues at specific sites to reduce access to proteases [244], which seems to have promising results. In order to translate CPPs to clinical research, however, there is a need to have a more robust targeted drug delivery system to ensure efficient delivery and drop off of the CPP and its cargo. In addition to this, there is a need to create robust CPPs that reach their specific target, minimising off-target delivery which could lead to adverse side effects [243].

## 7. Conclusions

PD and AD are progressive neurodegenerative disorders which represent a substantial unmet clinical need driving the requirement for new therapeutic approaches, focusing on both restorative and protective neuronal therapies. Extracellular vesicles have been tipped to be a leading therapy in this field, though they have some drawbacks in application as a sole therapeutic agent. These include cell target specificity, transport across the blood–brain barrier, and the ability to target multiple pathways of degeneration including oxidative stress, neuronal cell death and mitochondrial dysfunction. These limitations can potentially be overcome by combining the neuroprotective features of EVs, CPPs and miRNAs, which have complimentary features that lend themselves to being fit together in a novel combinational therapeutic agent able to tackle the multiple pathways of neurodegeneration exhibited by both AD and PD.
